# Differential Effects of Nitrogen and Phosphorus Fertilization Rates and Fertilizer Placement Methods on P Accumulations in Maize

**DOI:** 10.3390/plants13131778

**Published:** 2024-06-27

**Authors:** Sharifullah Sharifi, Songmei Shi, Hikmatullah Obaid, Xingshui Dong, Xinhua He

**Affiliations:** 1 National Base of International S&T Collaboration on Water Environmental Monitoring and Simulation in Three Gorges Reservoir Region, Chongqing 400716, China; nsharifullah@gmail.com (S.S.); hikmat_obaid@yahoo.com (H.O.); dxshui@email.swu.edu.cn (X.D.); 2 Centre of Excellence for Soil Biology, College of Resources and Environment, and Chongqing Key Laboratory of Plant Resource Conservation and Germplasm Innovation, School of Life Sciences, Southwest University, Chongqing 400716, China; 3 Department of Soil Science and Irrigation Management, Faculty of Plant Sciences, Afghanistan National Agricultural Sciences and Technology University (ANASTU), Kandahar 3801, Afghanistan; 4 School of Horticulture and Landscape, Yunnan Agricultural University, Kunming 650201, China; 2022020@ynau.edu.cn; 5 School of Biological Sciences, University of Western Australia, Perth 6009, Australia; 6 Department of Land, Air and Water Resources, University of California at Davis, Davis, CA 90616, USA

**Keywords:** broadcast, deep band, phosphorus accumulation, phosphorus use efficiency, side band, *Zea mays*

## Abstract

Crop production in Afghanistan suffers from limited phosphorus (P) availability, which severely hinders national agriculture sustainability. This study hypothesized that deep fertilizer placement could significantly enhance the uptake of immobile P and, thus, tissue P accumulation and crop yield. A two-year pot experiment growing two maize (*Zea mays*) hybrid cultivars (Xida-789 and Xida-211) was, therefore, conducted to test these hypotheses under three contrasting fertilizer placement methods (broadcast, side band, and deep band). In doing so, P concentrations in both maize tissues and soils were compared at 45, 60, and 115 days after sowing (DAS) under nine combinations of nitrogen (N) and P fertilizer rates (kg ha^−1^: N112P45, N112P60, N112P75, N150P45, N150P60, N150P75, N187P45, N187P60, N187P75). Results have shown that deep band placement significantly increased P uptake efficiency, leading to greater P concentration and accumulation in maize tissues compared to the other two fertilization methods. This improved P uptake was attributed to several factors associated with deep placement, including reduced P fixation, enhanced root access to P, and moisture availability for P uptake. Additionally, deep band placement combined with higher N application rates (N187 and N150) further enhanced plant P uptake by promoting P availability and utilization mechanisms. Deep band placement also resulted in significantly higher total soil P, Olsen-P, and P use efficiency than broadcast and side band methods, indicating a more efficient P fertilization strategy for maize that can improve growth and yield. This study also found positive correlations between P concentration in plant organs and soil Olsen-P, highlighting the importance of adequate soil P levels for optimal plant growth. Overall, our results have shown that deep band fertilizer placement emerged as a superior strategy for enhancing P uptake efficiency, utilization, and maize productivity compared to broadcast and side band placement. The outcome generated from the deep band fertilization by this greenhouse study can be recommended for field practices to optimize P fertilizer use and improve maize production while minimizing potential environmental P losses associated with broadcast fertilization.

## 1. Introduction

Phosphorus (P) is essential for various physiological processes in plants, including the constitution of phospholipids, nucleic acid, adenosine triphosphate, enzymes, and plant dry matter [1,2]. The application of P-containing fertilizers has been instrumental in increasing global food production, with significant contributions to higher agricultural productivity and global food security [3,4]. Globally, 86.6 Mt fertilizers were applied to cereal. These crops require greater N and P fertilizers during their life cycle to promote their growth and yield production. Cereal crops consume 55.9% N and 44.6% P globally, and out of them, maize (*Zea mays* L.) accounts for 17.8% N and 13.9% P, respectively [5,6]. Phosphorus plays significant roles in maize growth, root development, and formation of seed and fruit and provides resistance during periodic soil moisture deficits [7,8,9]. Maize accumulates P during the whole life cycle with maximum uptake between 3 and 6 weeks of growth. Furthermore, maize grain steadily accumulates P until maturity, and during the grain filling, considerable P translocation occurs from vegetative tissues to grain [7,10]. At its physiological maturity, maize accumulates 70 kg P ha^−1^ in the shoot and 23 and 40 kg P ha^−1^ in the straw and seed, respectively. In North America, 89 kg P ha^−1^ is required to produce 9.5 tons of maize grain [7]. Moreover, P fertilization increases soil Olsen-P and, thereby, increases shoot P concentration and photosynthesis rate. For instance, the net photosynthetic rate reached maximum when shoot P concentration increased from 2.0 to 2.4 g kg^−1^ under the critical 20.5 mg kg^−1^ soil Olsen-P [11]. Apart from the aforementioned roles, P deficiency has adverse effects on maize’ adventitious root and leaf growth, photosynthetic capacity, flower initiation, seed formation, and viability [4,12,13,14].

Maize is an important staple crop in Afghanistan after wheat and rice, with a yearly production of ~0.27 million tons from 0.14 × 10^6^ ha of plantation [15]. However, its productivity lags significantly behind the global average, at a 49% lower rate [16] due to various factors, including high soil pH, low P and nitrogen (N) availability, organic matter, soil moisture, fertilizer input, and poor field management [17,18,19]. While Afghanistan utilized a total of 7282 tons of P fertilizer (mostly diammonium phosphate) in 2021 to increase crop productivity [16], current application methods like broadcasting and shallow placement increase the risk of P stratification in the soil surface and soil erosion and water runoff transport concentrated P to water bodies and then eutrophication [13,20,21]. Phosphorus has to be rationally managed due to its limited and non-renewable nature [22,23]. Generally, plants can only absorb 10–25% of the P applied to soil in the same year, with the remainder becoming unavailable due to poor solubility, strong fixation to metal cation complexes, and slow diffusion in soil [1,24]. All of these have resulted in low P use efficiency and crop output, along with waterbody eutrophication and soil degradation [1,25].

Broadcast and deep band placement methods have been applied to study P accumulation in maize under different P fertilization rates [26,27] or a combination of varied N and P rates [28,29,30,31,32,33,34]. As a general rule, to reduce P losses on soil surface while improving P use efficiency, deep band placement of P fertilizer is recommended as it increases P availability in the root zone [20,21,35]. Applying fertilizer to moist soil also helps increase nutrient mobility and availability for plant uptake [9,36]. This is especially important in semi-arid environments, where high evaporation and temperature reduce soil moisture in the upper soil layer, resulting in restricted nutrient diffusion, lower P availability to root, reduced water use efficiency, and, consequently, lower crop growth rate and yield [37,38]. Additionally, placing chemical fertilizers in the soil subsurface could ensure that they remain in moist conditions for a longer period during the crop growth cycle [37,38].

This study hypothesized that deep fertilizer placement could increase (1) crop nutrient uptake, particularly immobile nutrients like P, and consequently, increase tissue P concentration and accumulation and (2) the fertilizer’s proximity to seeds and/or roots that could result in better utilization of fertilizer than broadcast or side band. These hypotheses were based on the fact that deep fertilizer placement could improve P fertilizer efficiency in soils with high P fixing capacity and provide nutrient access in the early growing season to crop roots for vigorous plant growth and development [39]. It also encourages root proliferation in nutrient-rich patches [37], while broadcast or shallow application reduces P availability to crop roots due to lower moisture in surface soil [36,40]. Importantly, Grant and Flaten [35] showed that deep fertilizer of NH_4_, urea, or (NH_4_)_2_SO_4_ along with P fertilizer increases P uptake compared to individual N or P applications. As a result, this study aimed to promote crop growth and productivity by determining (1) an ideal H_2_PO_4_^−^-P fertilization rate to integrate N application, (2) a rational fertilizer placement method by comparing the three proposed fertilizer placements of broadcasting, side banding, and deep banding, and (3) positive correlations between H_2_PO_4_ rates and placement methods.

## 2. Results

Since no significant differences had been observed, all presented data (means ± SE, *n* = 6) below for all measured parameters between two maize hybrid cultivars (Xida-789 and Xida-211) and two growth seasons (years 2018 and 2019) were first combined and then averaged under each corresponding fertilizer placement and fertilization rate. 

### 2.1. Leaf P Concentration and Accumulation

#### 2.1.1. Leaf P Concentration

**Figure 1:**
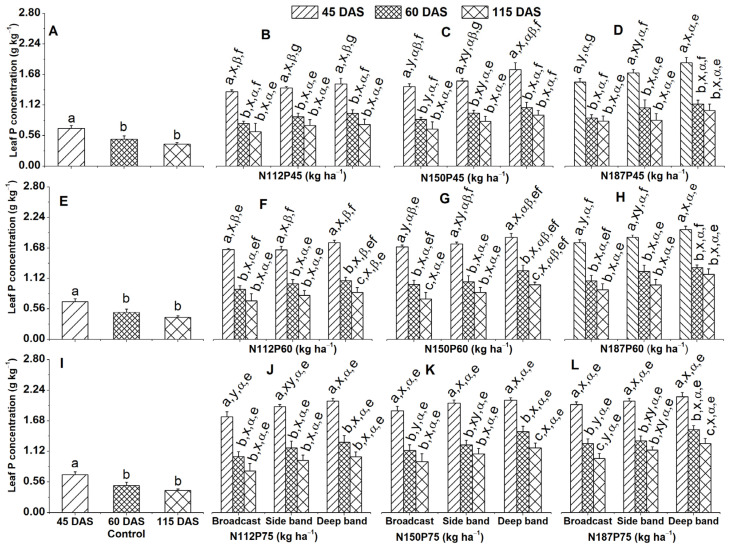
Effects of nitrogen and phosphorus fertilization rates and fertilizer placements on leaf P concentrations of maize at different growth days after sowing (DAS). Subfigures (**A**,**E**,**I**) are data at different DAS under the control treatment; (**B**–**D**), (**F**–**H**), or (**J**–**L**) represent data for the same P45, P60, or P75 fertilization rate under their corresponded different N rates and fertilizer placements. The letters (a, b, c) represent substantial differences among various growth days under constant N and P fertilization rate and constant fertilizer placement; the letters (x, y) denote significant differences between various fertilizer placement methods under constant growth days and constant N and P fertilization rates; the letters (α, β) represent significant differences between various N fertilization rates under constant P fertilization rate and constant fertilizer placement methods and plant growth days, and the letters (e, f, g) indicate significant differences between various P fertilization rates under constant N fertilization rate and constant fertilizer placement and plant growth days at *p* < 0.05.

Leaf P concentration (g kg^−1^) in the no-fertilization control was significantly higher at the V8 (45 DAS) growth stage than at the VT (60 DAS) and R6 (115 DAS) growth stages while being similar between the VT and R6 growth stages (Figure 1A,E,I).

Across various growth stages, leaf P concentration significantly differed under the same NP fertilization rate and fertilizer placement. That is to say, leaf P concentration was significantly greater at 45 DAS than at 60 DAS and 115 DAS for all the nine NP fertilization rates under all three placement methods (Figure 1B–D,F–H,J–L); the same trend was true between 6 DAS and 115 DAS except at N112P60 and N150P75 under deep side (Figure 1F,K) and at N150P60 and N187P75 under both broadcast and deep band (Figure 1G,L).

Among different fertilizer placements, deep band placement resulted in significantly higher leaf P concentration compared to broadcast placement under the combinations of N150P45, N187P45, N150P60, and N187P60 at 45 DAS, N187P75 at 60 DAS and 115 DAS, N112P75 at 45 DAS, and N150P75 at 60 DAS (Figure 1C,D,H,J–L).

Across all three placement methods (deep band, side band, and broadcast), leaf P concentration was significantly greater with N187 compared to N112 fertilization rate when combined with the same P45 fertilization rate at 45 DAS (Figure 1B–D). Similar results were observed for the P60 fertilization rate (Figure 1F–H).

Leaf P concentration increased with increasing P fertilization rates (P45, P60, P75) applied at the same N rate (N112, N150, N187). This effect was observed across various application methods and timings (Figure 1B–D,F–H,J–L). At 45 DAS, P75 resulted in significantly higher leaf P compared to P45 for all N rates and application methods (broadcast, side band, and deep band) except N187 under deep band (Figure 1B,C,F,G,J,K). P60 also showed a higher P concentration than P45 for N112 under broadcast, side, and deep band at 45 DAS, N150 under side band, and N187 under side band and broadcast (Figure 1B,C,F,G,J,K). At 60 DAS, under broadcast application, P75 maintained a significantly higher P concentration than P45 for all N rates (Figure 1B,F,J).

#### 2.1.2. Leaf P Accumulation

**Figure 2:**
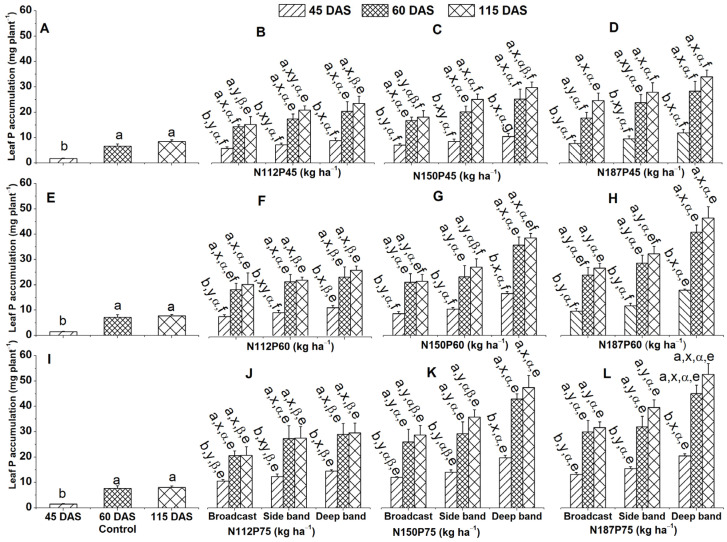
Effects of nitrogen and phosphorus fertilization rates and fertilizer placements on leaf P accumulations of maize at different growth days after sowing (DAS). Subfigures (**A**,**E**,**I**) are data at different DAS under the control treatment; (**B**–**D**), (**F**–**H**), or (**J**–**L**) represent data for the same P45, P60, or P75 fertilization rate under their corresponded different N rates and fertilizer placements. The letters (a, b) represent substantial differences among various growth days under constant N and P fertilization rate and constant fertilizer placement; the letters (x, y) denote significant differences between various fertilizer placement methods under constant growth days and constant N and P fertilization rates; the letters (α, β) represent significant differences between various N fertilization rates under constant P fertilization rate and constant fertilizer placement methods and plant growth days, and the letters (e, f) indicate significant difference between various P fertilization rates under constant N fertilization rate and constant fertilizer placement and plant growth days at *p* < 0.05.

The results showed a significant increase in leaf P accumulation (mg plant^−1^) over time across all fertilizer treatments (Figure 2A–L). Both the control group and P and N fertilized groups exhibited the highest leaf P accumulation at 115 DAS and 60 DAS compared to 45 DAS (Figure 2B–D,F–H,J–L).

Analysis of leaf P concentration revealed a clear pattern of higher accumulation with deep band placement compared to other methods. This was observed at 45 DAS for N112P45, N150P45, N187P45, N112P60, and N112P75 (Figure 2B–D,F,J), as was at 60 DAS for N18745 and 115 DAS for N112P45 (Figure 2B,D). Similarly, deep band placement led to higher P accumulation than side band and broadcast methods for N150P60, N187P60, N150P75, and N187P75 at 45 DAS, 60 DAS, or 115DAS (Figure 2G,H,K,L). At 115 DAS for N150P45, deep band and side band placements resulted in similar P levels (Figure 2C).

Increasing the N fertilization rates influenced leaf P accumulation in N187 and N150 more significantly than in N112 under the deep band at 45 DAS, 60 DAS, and 115 DAS and in N187 compared to N112 under the side band at 115 DAS (Figure 2F–H). At the P75 rate, leaf P accumulation was significantly greater in N187 compared to N112 under broadcast and side band placement at 45 DAS, 115 DAS, and across all harvest times (45, 60, and 115 DAS) under deep placement. Leaf P accumulation increased significantly with increasing P fertilizer rate (P75) compared to lower rates (P45 and P60) at 45 DAS for N112, N150, and N187 under side band and broadcast, N150 under side band, and N112 under deep band (Figure 2B–D,F–H,J–L). The same trend was also observed at 60 DAS for N112 and N187 under broadcast, at 115 DAS for N150 under broadcast and deep band, and for N187 under side band (Figure 2B–D,J–L).

### 2.2. Stem P Concentration and Accumulation

#### 2.2.1. Stem P Concentration

**Figure 3:**
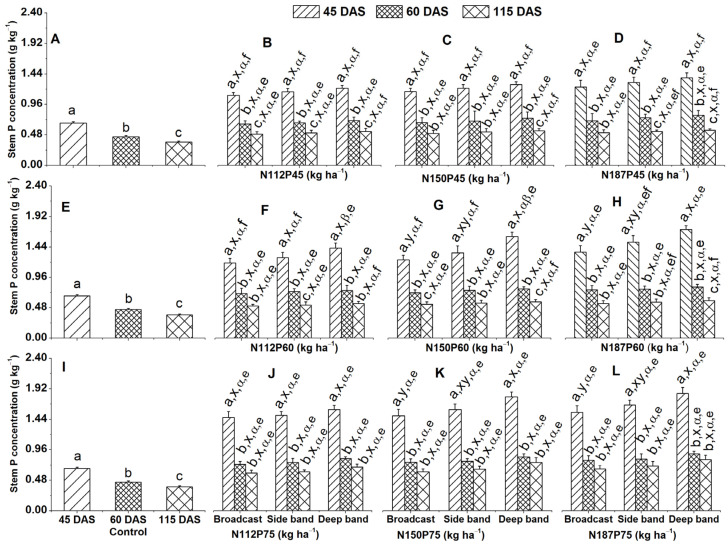
Effects of nitrogen and phosphorus fertilization rates and fertilizer placements on stem P concentration of maize at different growth days after sowing (DAS). Subfigures (**A**,**E**,**I**) are data at different DAS under the control treatment; (**B**–**D**), (**F**–**H**), or (**J**–**L**) represent data for the same P45, P60, or P75 fertilization rates under their corresponded different N rates and fertilizer placements. The letters (a, b, c) represent substantial differences among various growth days under constant N and P fertilization rates and constant fertilizer placement; the letters (x, y) denote significant differences between various fertilizer placement methods under constant growth days and constant N and P fertilization rate; the letters (α, β) represent significant differences between various N fertilization rates under constant P fertilization rate and constant fertilizer placement methods and plant growth days, and the letters (e, f) indicate significant differences between various P fertilization rates under constant N fertilization rate and constant fertilizer placement and plant growth days at *p* < 0.05.

Stem P concentration (g kg^−1^) showed a significant decline over time in the no-fertilization control treatment (Figure 3A,E,I). A similar trend was observed for all N and P fertilizer treatments and placement. Stem P concentration was significantly higher at 45 DAS compared to both 60 and 115 DAS for N112P75, N150P75, and N187P75 under deep band, side band, and broadcast applications (Figure 3J–L), as were N150P45 and N187P60 under side band and broadcast (Figure 3C,H), N187P45 under broadcast, N112P60 under deep band and broadcast, N150P60 under side band (Figure 3D,F,G), N112P45 under all placement methods, N150P45 and N187P60 under deep band (Figure 3B,C,H), N187P45 under side band and deep band, N112P60 under side band, and N187P60 under deep band (Figure 3D,F,H).

The effect of fertilizer placement (deep band, side band, and broadcast) on stem P concentration was significantly higher in deep placement compared to broadcast for N150P60, N187P60, N150P75, and N187P75 treatments at 45 DAS (Figure 3G,H,K,L). Similarly, N fertilization rates (N112, N150, N187) showed a significantly greater stem P concentration with N187 compared to those receiving N112 for the same P60 treatment under deep placement (Figure 3B–D,F–H,J–L).

Stem P concentration increased significantly with increasing P fertilization rates (P75 > P60 > P45) at specific N rates (N112 and N150) and placement methods. This effect was observed at 45 DAS under broadcast and side band applications (Figure 3B,C,F,G,J,K) and at 115 DAS under deep band application for N112, N150 and N187 (Figure 3D,H,L).

#### 2.2.2. Stem P Accumulation

**Figure 4:**
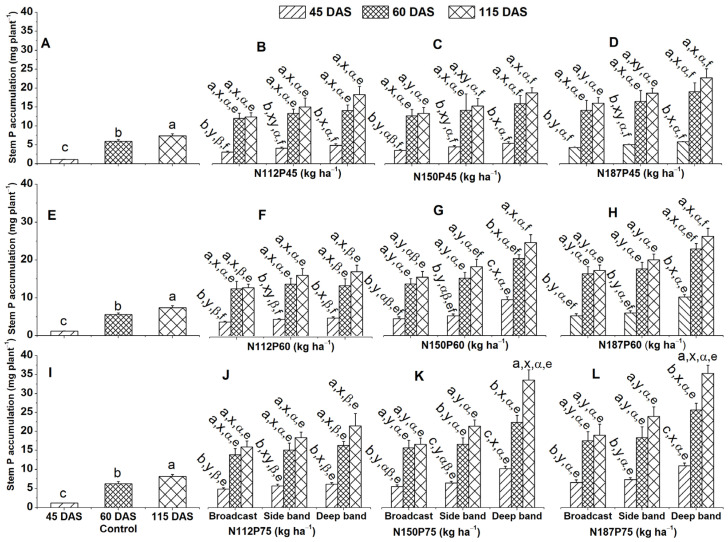
Effects of nitrogen and phosphorus fertilization rates and fertilizer placements on stem P accumulation of maize at different growth days after sowing (DAS). Subfigures (**A**,**E**,**I**) are data at different DAS under the control treatment; (**B**–**D**), (**F**–**H**), or (**J**–**L**) represent data for the same P45, P60, or P75 fertilization rate under their corresponded different N rates and fertilizer placements. The letters (a, b, c) represent substantial differences among various growth days under constant N and P fertilization rates and constant fertilizer placement; the letters (x, y) denote significant differences between various fertilizer placement methods under constant growth days and constant N and P fertilization rates; the letters (α, β) represent significant differences between various N fertilization rates under constant P fertilization rate and constant fertilizer placement methods and plant growth days, and the letters (e, f) indicate significant difference between various P fertilization rates under constant N fertilization rate and constant fertilizer placement and plant growth days at *p* < 0.05.

Maize grown without fertilizer (control group) showed the highest stem P accumulation (mg plant^−1^) at 115 DAS, followed by 60 DAS and then 45 DAS (Figure 4A,E,I).

Across all fertilization regimes (N and P rates and placement methods), stem P accumulation was significantly higher at 115 DAS and 60 DAS compared to 45 DAS. This pattern was observed for N112P45, N150P45, N187P45, N112P60, N187P60, N112P75 under the deep band, side band, and broadcast (Figure 4B–D,F,H,J), for N150P60 and N187P75 under broadcast and side band (Figure 4G,L), and for N150P75 under broadcast (Figure 4K). For N150P60 and N187P75 fertilized maize, deep band application resulted in significantly higher stem P accumulation at 115 DAS compared to 60 DAS, which, in turn, was higher than 45 DAS (Figure 4G,K,L). A similar trend was also observed for N150P75 with side band and deep band placements.

Deep band placement resulted in significantly greater stem P accumulation compared to side band and broadcast applications under N150P60, N187P60, N150P75, and N187P75 at 45 DAS, 60 DAS, or 115 DAS (Figure 4G,H,K,L). A similar trend was also observed for N112P45, N112P60, and N112P75 at 45 DAS (Figure 4B,F,J), and for N150P45 and N187P45 at 45 DAS and 115 DAS (Figure 4C,D).

Among different N rates, stem P accumulation was significantly greater at N187 than N112 for the same P45 under broadcast at 45 DAS; the P60 accumulation was the same under broadcast at 45 DAS and 115 DAS and under side band at 45 DAS; and P75 was the same under broadcast and side band at 45 DAS (Figure 4B,D,F,H,J,L). Moreover, stem P accumulation was significantly higher at N187 and N150 than N112 for the same P60 under a deep band at 45 DAS, 60 DAS, or 115 DAS, and P75 was the same under a deep band at 45 DAS, 60 DAS, or 115 DAS (Figure 4B–D,F–H,J–L).

For maize receiving the same amount of N fertilizer, those that received the highest P rate (P75) accumulated more P in their stems compared to those receiving lower P rates (P45 and P60) at 45 DAS. This trend was observed across all fertilizer placement methods (broadcast, side band, and deep band) for N150 and N187 (Figure 4C,D,G,H,K,L). Similarly, for N112, P75 resulted in greater stem P accumulation compared to P45 across all placement methods at 45 DAS (Figure 4B,F,J).

### 2.3. Root P Concentration and Accumulation

#### 2.3.1. Root P Concentration

**Figure 5:**
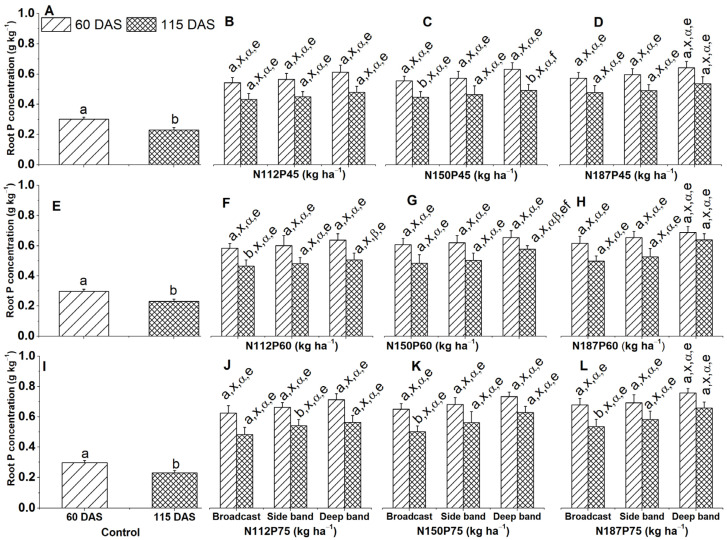
Effects of nitrogen and phosphorus fertilization rates and fertilizer placements on root P concentration of maize at different growth days after sowing (DAS). Subfigures (**A**,**E**,**I**) are data at different DAS under the control treatment; (**B**–**D**), (**F**–**H**), or (**J**–**L**) represent data for the same P45, P60, or P75 fertilization rates under their corresponded different N rates and fertilizer placements. The letters (a, b) represent substantial differences among various growth days under constant N and P fertilization rates and constant fertilizer placement; the letters (x) denote significant differences between various fertilizer placement methods under constant growth days and constant N and P fertilization rate; the letters (α, β) represent significant differences between various N fertilization rates under constant P fertilization rate and constant fertilizer placement methods and plant growth days, and the letters (e, f) indicate significant difference between various P fertilization rates under constant N fertilization rate and constant fertilizer placement and plant growth days at *p* < 0.05.

Maize grown without fertilizer (control) showed higher concentrations of P (g kg^−1^) in their roots at 60 DAS than at 115 DAS (Figure 5A,E,I). Likewise, higher root P concentration at 60 DAS was also observed for several combinations, namely, N112P60, N150P75, and N187P75 under broadcast placement (Figure 5C,K,L), N150P45 under broadcast and deep bands (Figure 5C), and N112P60 under broadcast (Figure 5F).

N fertilizer rate (N112, N150, or N187) also had no significant impact on root P concentration, regardless of the P rate (P45, P60, or P75) at both 60 and 115 DAS. The only exception was a higher concentration observed in N187 compared to N112 for the same P60 rate under deep band placement at 115 DAS (Figure 5F,H). P fertilizer rate (P45, P60, and P75) also did not affect root P concentration when applied with the same N rate (N112, N150, or N187) and placement method at 60 and 115 DAS. The sole exception was a significantly greater concentration in P75 compared to P45 for N150 under a deep band at 115 DAS (Figure 5B–D,F–H,J–L).

#### 2.3.2. Root P Accumulation

**Figure 6:**
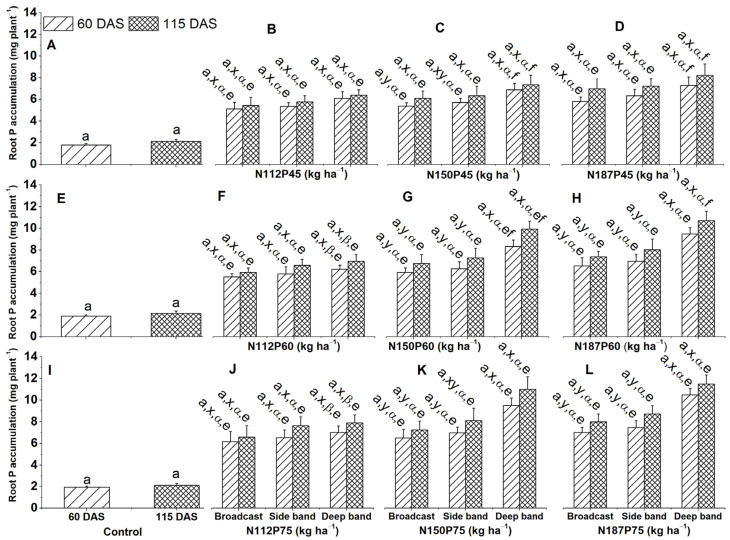
Effects of nitrogen and phosphorus fertilization rates and fertilizer placements on root P accumulation of maize at different growth days after sowing (DAS). Subfigures (**A**,**E**,**I**) are data at different DAS under the control treatment; (**B**–**D**), (**F**–**H**), or (**J**–**L**) represent data for the same P45, P60, or P75 fertilization rate under their corresponded different N rates and fertilizer placements. The letters (a) represent substantial differences among various growth days under constant N and P fertilization rates and constant fertilizer placement; the letters (x, y) denote significant differences between various fertilizer placement methods under constant growth days and constant N and P fertilization rates; the letters (α, β) represent significant differences between various N fertilization rates under constant P fertilization rate and constant fertilizer placement methods and plant growth days, and the letters (e, f) indicate significant difference between various P fertilization rates under constant N fertilization rate and constant fertilizer placement and plant growth days at *p* < 0.05.

Deep band placement significantly enhanced greater maize root accumulation compared to the side band or broadcast placements for N150P60 and N187P60 at 60 DAS and 115 DAS, N150P75 at 60 DAS, N187P75 at 60 DAS and 115 DAS (Figure 6G,H,K,L), N150P45 at 60 DAS and N150P75 at 115 DAS (Figure 6C,K). The combination of N187 and N150 rates with P75 and P60 resulted in higher root P accumulation under deep band placement at 60 and 115 DAS (Figure 6F–H,J–L).

Greater root P accumulation was enhanced under P75 compared to P45 for the same N150 at both 60 and N150 DAS and for N187 at 115 DAS (Figure 6C,D,K,L). In addition, P75 and P60 resulted in higher root P accumulation compared to P45 for the same N187 at 60 DAS (Figure 6D,H,L).

### 2.4. Seed P Concentration and Accumulation

#### 2.4.1. Seed P Concentration

**Figure 7:**
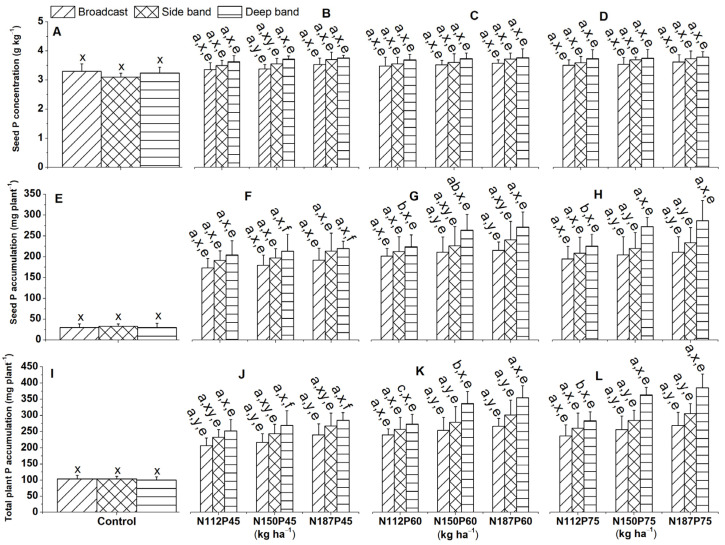
Effects of nitrogen and phosphorus fertilization rates and fertilizer placement on seed P concentrations (**A**–**D**), seed P accumulations (**E**–**H**), and total plant (leaf + stem + seed + root) P accumulations of maize crop at the harvest (**I**–**L**). The letters (a, b) represent substantial differences between various N fertilization rates under constant P fertilization rate and constant fertilizer placement; the letters (x, y) indicate significant differences between fertilizer placements for the same NP fertilization rate, and the letters (e, f) represent significant difference between various P fertilization rates under constant N fertilization rate and constant fertilizer placement methods at *p* < 0.05.

In pots with no fertilizer, seed P concentration did not differ significantly between the deep band, side band, and broadcast placements at 115 DAS (Figure 7A). When fertilizer was applied, seed P concentration was significantly higher with deep band placement compared to broadcast placement for the lower N and P rates (N112P45 and N150P45) (Figure 7B).

#### 2.4.2. Seed P Accumulation

Deep band placement resulted in significantly higher seed P accumulation compared to side band and broadcast application for the same N150P60, N187P60, N150P75, and N187P75 fertilization rates (Figure 7G,H).

### 2.5. Total plant P Accumulation

Deep placement resulted in significantly greater total plant P accumulation (sum of leaf, stem, seed, and root) compared to broadcast for most fertilizer combinations (N150P45, N187P45, N112P60, N112P75) (Figure 7J–L). Interestingly, total plant P accumulation was significantly higher under deep band placement compared to side band and broadcast for fertilizer combinations of N150P75 and N187P75 (Figure 7L).

Total plant P accumulation was significantly greater in N187 than N112 with the same P60 and P75 under side band (Figure 7K,L), while significantly greater total P accumulation was produced in N187, followed by N150, and it was lower at N112 for the P60 under deep band (Figure 7K).

Total plant P accumulation among P rates (P45, P60, P75) was significantly greater in P75 than P45 for the same N150 under broadcast and side band, as well as for the same N187 under the side band (Figure 7J–L). However, significantly greater total P accumulation was produced in P75, followed by P60, and it was lower in P45 for the same N150 and N187 under the deep band (Figure 7J–L).

### 2.6. Total Soil P Concentration

**Figure 8:**
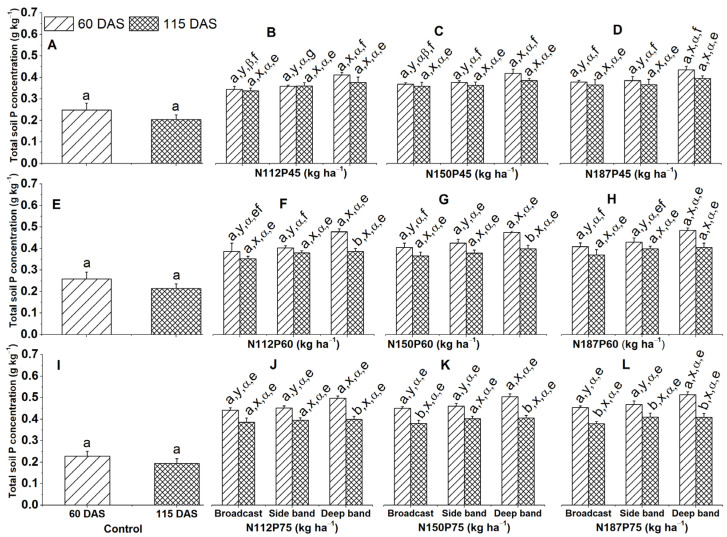
Effects of nitrogen and phosphorus fertilization rates and fertilizer placements on total soil P concentration at different growth days after sowing (DAS). Subfigures (**A**,**E**,**I**) are data at different DAS under the control treatment; (**B**–**D**), (**F**–**H**), or (**J**–**L**) represent data for the same P45, P60, or P75 fertilization rates under their corresponded different N rates and fertilizer placements. The letters (a, b) represent substantial differences among various growth days under constant N and P fertilization rates and constant fertilizer placement; the letters (x, y) denote significant differences between various fertilizer placement methods under constant growth days and constant N and P fertilization rates; the letters (α, β) represent significant differences between various N fertilization rates under constant P fertilization rate and constant fertilizer placement methods and plant growth days, and the letters (e, f, g) indicate significant differences between various P fertilization rates under constant N fertilization rate and constant fertilizer placement and plant growth days at *p* < 0.05.

Total soil P concentration (g kg^−1^) showed varied responses to fertilization rate, placement methods, and sampling day. However, total soil P concentration between various growth days under constant N and P fertilization rates and constant fertilizer placement methods were considerably greater at 60 DAS than at 115 DAS for N112P60, N150P60, and N112P75 under the deep band, also for N187P75 under the deep band, side band, and broadcast (Figure 8F,G,J,L).

At 60 DAS, under all NP fertilizer combination rates, total soil P concentration was significantly greater in the deep band compared to the side band and broadcast placements (Figure 8C,D,F–H,J–L).

Total soil P concentration among P rates (P45, P60, P75) was significantly increased at P75 and P60 than P45 for the same N112, N150, and N187 under deep band at 60 DAS (Figure 8B–D,F–H,J–L), as well as at P75 than P45 for the same N112 and N187 under broadcast and side band at 60 DAS (Figure 8B,D,F,H,J,L) and at P75 than P60 and P45 for the same N150 and N187 under broadcast at 60 DAS (Figure 8D,G,H,K,L).

### 2.7. Olsen-P

**Figure 9:**
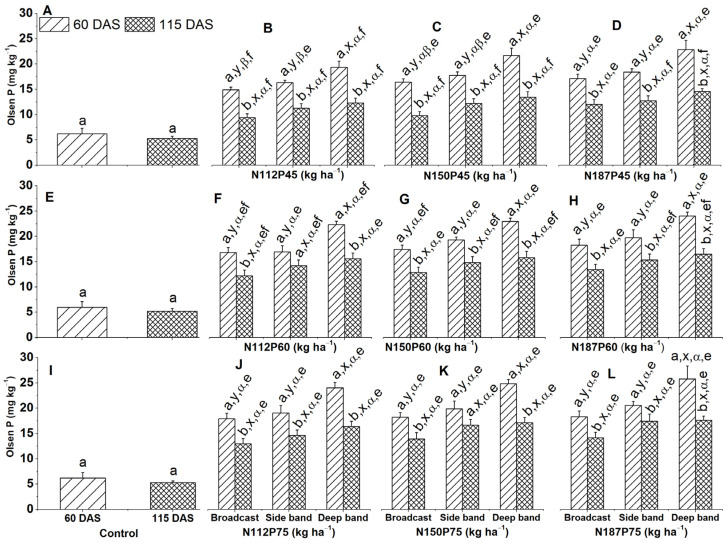
Effects of nitrogen and phosphorus fertilization rates and fertilizer placements on Olsen-P concentration at different growth days after sowing (DAS). Subfigures (**A**,**E**,**I**) are data at different DAS under the control treatment; (**B**–**D**), (**F**–**H**), or (**J**–**L**) represent data for the same P45, P60, or P75 fertilization rate under their corresponded different N rates and fertilizer placements. The letters (a, b) represent substantial differences among various growth days under constant N and P fertilization rate and constant fertilizer placement; the letters (x, y) denote significant differences between various fertilizer placement methods under constant growth days and constant N and P fertilization rates; the letters (α, β) represent significant differences between various N fertilization rates under constant P fertilization rate and constant fertilizer placement methods and plant growth days, and the letters (e, f) indicate significant differences between various P fertilization rates under constant N fertilization rate and constant fertilizer placement and plant growth days at *p* < 0.05.

Across all fertilization rates and placements, Olsen-P was significantly higher at 60 DAS compared to 115 DAS, as observed in N112P45, N150P45, N187P45, N150P60, N187P60, N112P75, and N187P75 under the deep band, side band, and broadcast (Figure 9B–D,G,H,J,L). A similar trend was also observed for N112P60 and N150P75 under broadcast and deep band (Figure 9F,K).

When comparing fertilizer placements with the same N and P rates, deep band application resulted in significantly higher Olsen-P levels at 60 DAS compared to side band and broadcast applications. This was observed for N112P45, N150P45, N187P45, N112P60, N150P60, N187P60, N112P75, N150P75, and N187P75 at 60 DAS (Figure 9B–D,F–H,J–L). At 115 DAS, deep band placement showed higher Olsen-P compared to broadcast application for N112P45, N150P45, and N112P75 (Figure 9B,C,J).

Olsen-P levels in the soil were significantly higher in N187 compared to N112 when applied with the same P45 under broadcast and side band placement at 60 DAS (Figure 9B,D).

On the other hand, P fertilization rates significantly influenced Olsen-P levels. When the N rate and placement method were constant, Olsen-P was significantly higher with the P75 application compared to P45 (Figure 9B,F,J). This was observed for N112 under broadcast at both 60 and 115 DAS, under the side band at 115 DAS, and under the deep band at 60 DAS. Similarly, N150 and N187 also showed higher Olsen-P with P75 compared to P45 under the deep band, side band, and broadcast at 115 DAS (Figure 9C,D,F–H,L).

### 2.8. Phosphorus Use Efficiency

Deep fertilizer placement significantly enhanced P uptake by the crop. This was evident in greater phosphorus agronomy efficiency (PAE) and partial factor productivity for applied P (PFP_P_) compared to broadcast and side band fertilizations, particularly under N150P75 and N187P75, and phosphorus use efficiency (PUE) under N150P60, N187P60, N150P75, and N187P75. Furthermore, for the same P rate and placement method, increasing N rates from N112 to N150 or N187 significantly enhanced both PUE and PFP_P_ under deep band placement; this trend was not observed with broadcast or side band methods.

Interestingly, PFPP among different P fertilization rates for all placement methods was significantly greater under P45 compared to P60 or P75 for the same N150 and N187 fertilization rates.

### 2.9. Relationships between Plant Tissue P Accumulations and Concentrations or between Tissue P Concentrations or Accumulations and Soil Olsen-P Concentrations

**Figure 10:**
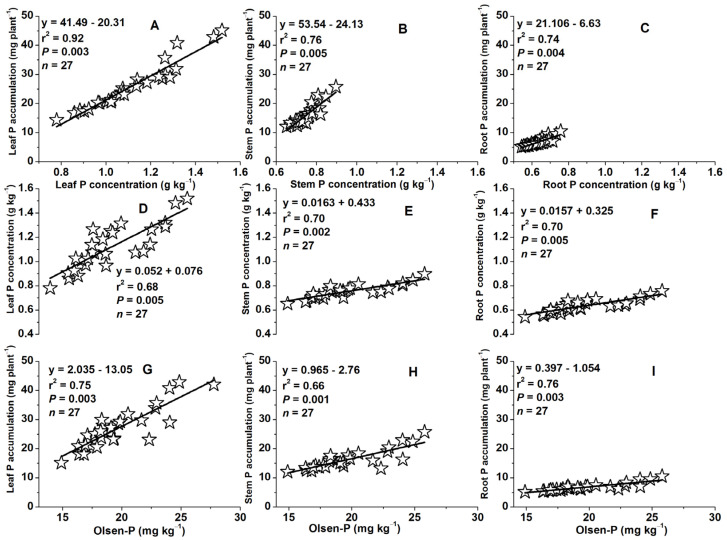
Relationships between tissue P concentrations and accumulations in leaf (**A**), stem (**B**), or root (**C**) at the VT (tasseling, at 60 days after sowing) growth stage, also between tissue P concentrations or P accumulations in leaf (**D**,**G**), stem (**E**,**H**), root (**F**,**I**), and Olsen-P concentrations at 60 days after sowing.

Plant tissue phosphorus (P) concentration and accumulations showed strong positive correlations in leaves, stems, and roots (*r*^2^ = 0.92–0.76–0.74, *P* = 0.003–0.005–0.004) at the VT (tasseling, at 60 DAS) growth stage (Figure 10A–C). Similarly, P concentrations and accumulations in each tissue (leaf, stem, and root P) were positively related to the available soil phosphorus (Olsen-P) at 60 DAS (*r*^2^ = 0.68–0.70–0.70, 0.75–0.66–0.76, *P* = 0.005–0.002–0.005, 0.003–0.001–0.003) (Figure 10D–F,H–I).

**Figure 11:**
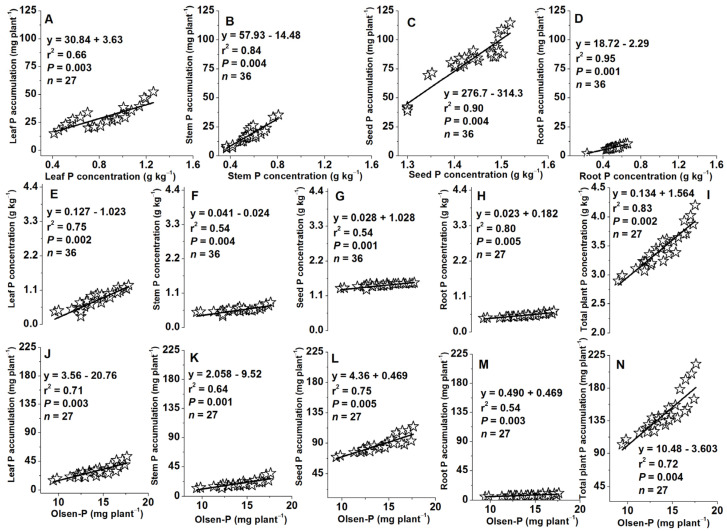
Relationships between tissue P concentration and accumulation in leaf (**A**), stem (**B**), seed (**C**), and root (**D**) at the R6 (physiological maturity or harvest at 115 days after sowing), between tissue P concentration in leaf (**E**), stem (**F**), seed (**G**), root (**H**), total plant (leaf + stem + seed + root; **I**), and soil Olsen-P at 115 DAS and between tissue P accumulation in leaf (**J**), stem (**K**), seed (**L**), root (**M**), total plant (leaf + stem + seed + root; **N**), and soil Olsen-P at 115 days after sowing.

At harvest (115 DAS), positive correlations were also observed between P concentration and accumulation in leaves, stems, seeds, and roots (r^2^ = 0.66–0.84–0.90–0.95, *P* = 0.003–0.004–0.004–0.001, Figure 11A–D). Likewise, Olsen-P concentration in the soil showed positive relationships with P concentrations in all plant tissues (leaf, stem, seed, root) and total plant (leaf + stem + seed + root) at 115 DAS (r^2^ = 0.75–0.54–0.54–0.80–0.83; *P* = 0.002–0.004–0.001–0.005–0.002, Figure 11E–I). Finally, Olsen-P concentration also showed positive correlations with P accumulation in all plant tissues and total plant at 115 DAS (r^2^ = 0.71–0.64–0.75–0.54–0.72; *P* = 0.003–0.001–0.005–0.003–0.004, Figure 11J–N).

## 3. Discussions

Deep fertilizer placement fulfills the moisture requirement for the nutrient dissolution process in the deeper soil layer and, in turn, increases nutrient availability for root uptake by maize plants and prevents nutrient deficiencies [41]. Studies have shown that placing N and P fertilizers near maize seeds enhances their synergistic interaction, increases nutrient concentration and P uptake, and promotes growth and development, nutrient use efficiency, and overall productivity, ultimately leading to higher seed yields [9,42]. To verify these potential growth benefits, a two-year (2018 and 2019) greenhouse pot experiment was conducted to examine nine different combinations of N and P fertilization rates (N112P45, N112P60, N112P75, N150P45, N150P60, N150P75, N187P45, N187P60, and N187P75 kg ha^−1^) applied using three placement methods (deep band, side band, and broadcast), and the agronomic performance of two maize hybrid cultivars (Xida-789 and Xida-211) were investigated.

### 3.1. Greater P Concentration under Deep Band

Deep band fertilizer placement significantly enhanced phosphorus (P) uptake compared to broadcast placement. This was evident in increased leaf P concentration by 9.6% and 10.2% under N150P45 and N187P45, respectively, at 45 DAS, 13.7% and 9.9% under N150P75 and N187P75 at 60 DAS, and by 12.1% under N187P75 at 115 DAS (Figure 1C,D,K,L). Similarly, stem P concentration also showed a significant increase (12.9% and 11.6% under N150P60 and N187P60, respectively) with deep banding at 45 DAS (Figure 3G,H,K,L). Seed P concentration also increased by 3.4% and 4.8% under N112P45 and N150P45 (Figure 7B). These results indicate that deep banding of fertilizer, which is placed in close proximity to root systems, enhances the interaction between the fertilizer and roots, thereby optimizing P uptake efficiency [28,43]. Conversely, broadcasting exposes fertilizers to a larger soil surface area, leading to substantial losses through adsorption, leaching, and erosion [44]. This can cause P deficiency in the early growth cycle, which will have a negative impact on both the crop’s growth and its reproductive development [35]. Even if adequate P levels are reached later, they may not fully compensate for the initial deficiency. Deep placement, on the other hand, minimizes these losses and ensures a readily available P source within the root zone throughout the whole growth season [35,45].

The current study showed that higher P fertilization rates (P60 and P75) significantly increased leaf and stem P concentrations compared to the lower rate (P45) regardless of NP rate combination and fertilizer placement (Figure 1D,H,L; Figure 3B,C,F,G,J,K). These results suggest that P fertilization rates at P75 or P60 are optimal for enhancing P uptake in maize plants under the given soil conditions. This aligns with the established soil P recommendations for maize, where medium soil P levels (similar to those at 16.3 and 15.1 mg kg^−1^ in 2018 and 2019 in our study, respectively) necessitate P application rates between 60 and 100 kg ha^−1^ [44]. By meeting maize P demand throughout the growth season, these optimal P fertilization rates promote plant growth, seed yield, N and P concentrations, and accumulation in various maize organs.

The findings from this study corroborate previous studies demonstrating that higher P fertilization rates enhance maize P accumulation and biomass production. For example, Nadeem et al. [46] observed increased grain dry weight and P accumulation in maize fertilized with P92.50 kg ha^−1^ compared to P5.60 kg P ha^−1^. In addition, Ahmad et al. [47] demonstrated that P fertilization at P90 kg ha^−1^ significantly enhanced grain ear^−1^, 1000-seed weight, harvest index, seed yield, and total biomass compared to P60 kg ha^−1^.

### 3.2. Greater Soil Total P and Olsen-P Concentrations under Deep Band

In the present study, deep band placement resulted in significantly higher total soil phosphorus concentration compared to broadcast and side band placements across all N and P fertilization rates (N112P45, N150P45, N187P45, N112P60, N150P60, N187P60, N112P75, N150P75, and N187P75 (Figure 8B,C,F–H,J–L). This observation was mirrored by higher Olsen-P concentration at 60 DAS (Figure 9B,C,F–H,J–L).

Deep fertilizer placement offers several advantages that contribute to increased soil P concentration. Firstly, it minimizes fertilizer contact with soil particles, thereby reducing P fixation compared to broadcast application [48,49]. In calcareous soil specifically, banding P fertilizer reduces contact between fertilizer and soil while maximizing root-fertilizer contact and P concentration compared to broadcasting, leading to less immobilization and greater P uptake [50]. Secondly, deep placement aligns fertilizer with the zone of adequate moisture in subsurface soil, which is crucial for P solubility. This promotes a significant increase in soil inorganic P fractions like dicalcium phosphate (Ca2-P), octacalcium phosphorus (Ca8-P), aluminum phosphorus (Al-P), and iron phosphorus (Fe-P) at various soil depths [36]. Conversely, P broadcasting induces P stratification near the soil surface. This increases P losses through mechanisms like soil and wind erosion and surface runoff, ultimately reducing P availability for plant uptake. Compared to broadcasting, injecting fertilizers like monoammonium phosphate and poultry litter 1 cm below the soil surface has been shown to significantly reduce P loss by 98% and 84%, respectively [20].

Our findings showed that higher P fertilization rates (P60 and P75) significantly increased both total soil P and Olsen-P concentrations compared to the lower rates (P45) at various N fertilization levels and fertilizer placement methods (deep band, side band, and broadcast) at 60 and 115 days after sowing (DAS) (Figure 8 and Figure 9). These results indicate that P availability in the soil increases with higher P application rates. Studies have shown a positive correlation between P fertilizer application rates and soil P availability. For instance, P100 and P200 P_2_O_5_ kg ha^−1^ considerably increase soil available P by 100% and 200%, respectively, compared to no-P control [51]. A 21-year long-term study showed that P20, P39, P59, and P79 kg P ha^−1^ considerably increased Olsen-P by 3.7, 5.2, 11.2, and 20.6 mg P kg^−1^ and total soil P concentration by 4.2%, 26.0%, 36.5%, and 49.8%, respectively [52].

Total soil P concentration and Olsen-P concentration at the tasseling stage (60 DAS) were significantly different among fertilizer placement methods and between N and P fertilizer combination rates. However, Olsen-P and total soil P concentrations at the harvest stage (115 DAS) remained constant among fertilizer placement methods and between different N and P fertilization rates. Maize accumulates a major amount of P during the initial growth stages from 3 to 6 weeks [7,10]; as the plant growth progresses until maturity, the applied P in soil is either taken up by plant roots or fixed to soil particles so that both Olsen-P and total soil P concentrations are decreased at the maturity stage.

In this study, deep fertilizer placement creates a nutrient-concentrated zone in the immediate vicinity of maize root, which promotes nutrient uptake, improves efficient nutrient utilization, and minimizes P contact with soil, resulting in less phosphorus sorption and precipitation reactions and, thus, enhanced availability to crops. This approach has also reserved applied P in a small, localized area for a longer period, which has been utilized by plant roots in later growth stages. Moreover, in this study, placing N and P fertilizer together near maize seed increased their synergistic interaction, nutrient concentration, and P uptake, promoted growth and root development, increased nutrient use efficiency and plant productivity, and produced higher seed yield.

### 3.3. Greater P Accumulation and Yield Production under Deep Band

This study found that deep band application significantly increased P accumulation in maize leaves, stems, and roots compared to sideband and broadcast application at all time points (45, 60, 115 DAS) and for N150P60, N187P60, N150P75, and N187P75 (Figure 2G,H,K,L; Figure 4G,H,K,L; Figure 6G,H,K,L). Deep band application also improved seed P accumulation under N150P75 and N187P75 (Figure 7H) and higher total plant P accumulation under N150P60 and N187P60 (Figure 7K). These findings can be explained by several factors. First, the broadcasting fertilizer mixes it with a larger soil volume, exposing it to a greater surface area. However, this can lead to P stratification near the soil surface, which increases the risk of immobilization or loss through runoff [44]. This, in turn, reduces the amount of P available for plant uptake and potential P deficiency. Deep band placement, on the other hand, minimizes soil–fertilizer contact, thereby improving the availability of P for plant uptake [44,53,54,55].

Second, P is relatively immobile in soil and relies on diffusion for uptake by plant roots. Placing fertilizer close to the roots provides them with easier access to this essential nutrient, leading to greater P-use efficiency compared to broadcasting [44,56]. Third, for efficient P uptake, plants require a sufficient P supply during the early growth stages to develop a robust root system with enhanced P acquisition capabilities [57]. Studies by Hopkins [58] and Dhillon et al. [56] showed that applying the required P fertilizer near the seeds at sowing is a more efficient strategy than broadcasting or split applications. Deep band placement not only fulfills this requirement but also ensures access to moisture in deeper soil layers, which can further enhance P solubility and mobility in the root zone. This promotes root proliferation, N and P uptake, and P use efficiency [45,59,60].

Phosphorus accumulation in maize is also enhanced with higher N rates (N187 and N150) compared to a lower rate (N112) with the same P level (P60 or P75) under deep band placement. This was observed in leaves and stems at various points throughout the growing season (45 DAS, 60 DAS, or 115 DAS) (Figure 2F–H,J–L; Figure 4F–H,J–L; Figure 6F–H,J–L). Root P accumulation also increased at 60 and 115 DAS (Figure 2F–H,J–L; Figure 4F–H,J–L; Figure 6F–H,J–L). Furthermore, total plant P accumulation at harvest (115 DAS) and seed P accumulation at the same P level (P75) under deep band placement were significantly greater with higher N rates (Figure 7H,K,L). These findings suggest a synergistic effect of N fertilization on P uptake by maize. Studies have shown that N application enhanced plant P availability and utilization, including stimulating the conversion of organic P forms into inorganic P forms that are more readily taken up by plants [60], reduce the amount of soil iron (Fe) and aluminum (Al) oxides, which are known to adsorb P and make it unavailable to plants [61], and improve P utilization efficiency, P translocation, and P translocation efficiency [60]. A field experiment with maize in Zimbabwe demonstrated that increasing N application rates from 0, 40, 80 to 120 N kg ha^−1^ led to higher total plant and grain P accumulation [62].

### 3.4. Greater P Use Efficiency under Deep Band

This study found that deep fertilizer placement significantly increased phosphorus agronomy efficiency (PAE), partial factor productivity for the applied phosphorus (PFP_P_), and phosphorus use efficiency (PUE) compared to broadcast and side band fertilization (Table 1). This aligns with previous research demonstrating that placing P closer to the seed or root zones enhances PUE while reducing P application rates compared to broadcasting [39,63]. Phosphorus is a relatively immobile nutrient in the soil and readily fixes to soil particles, especially when broadcast on the soil surface. Deep fertilizer, however, reduces P fixation reactions due to spatial availability, which enhances the acquisition of P and, consequently, P recovery efficiency [39,60]. Studies report that an average PUE is typically only around 5–10% under broadcast fertilization but considerably increases to 30–35% under deep placement [64].

### 3.5. Variations in Relationships between Concentrations of Tissue P, Soil P, and Plant P Accumulations

In this study, positive relationships were observed between P concentrations and accumulations in leaves, stems, seeds, roots, and total plant biomass at 60 DAS and 115 DAS (Figure 10A–C and Figure 11A–D). Similarly, P concentrations and accumulations in these plant parts also showed a positive relationship with Olsen-P concentrations at 60 DAS and 115 DAS (Figure 10D–F,G–I and Figure 11E–I,J–N). These results are consistent with previous studies that have documented positive relationships between plant organ P content and soil-available P. For instance, ref. [65] reported strong correlations between soil P and P concentrations in leaves, stems, roots, and fruits of orange trees. They further suggested that fertilizer application strategies could be optimized based on relationships between soil and plant P concentrations rather than relying solely on soil or plant P levels. Furthermore, Liu et al. [40] observed that increased P fertilization led to a higher proportion of inorganic P fractions (Ca2-P, Ca8-P, Al-P, and Fe-P) in winter wheat, which positively correlated with seed yield (ranging from 67% to 86%) and aboveground biomass (21% to 41%). Fan et al. [61] also observed significant correlations between various soil P fractions (available P, labile P, moderately labile P) and root biomass/length density in a subtropical forest ecosystem. The strongest correlations were found between available P and both root biomass (42%) and root length density (55%).

Maize takes up most P in its early growth between 3 and 6 weeks. This uptake generally happens alongside the accumulation of dry matter, though P uptake tends to be slightly ahead during this early stage but declines at later growth stages [7]. This pattern highlights that a positive correlation between seed yield or biomass production and P accumulation in plant organs indicates efficient internal P utilization by wheat and lupin [66]. Ma et al. [67] further demonstrated a strong correlation between root P concentration and root biomass (84%) and leaf P concentration and leaf biomass (91%).

## 4. Materials and Methods

### 4.1. Study Site and Plant Materials

This 2-year pot study was conducted over two growing seasons from 29 May to 15 September 2018, and 15 May to 28 August 2019, in a glass greenhouse located at the National Monitoring Base for Purple Soil Fertility and Fertilizer Efficiency (NMBPSFFE), Southwest University (29°48′ N, 106°24′ E, 266.3 m asl), Chongqing, China. The rain-shed greenhouse had natural light and temperature and was constructed with rain-proof glasses.

The region experiences a humid subtropical climate, with mean summer temperatures of 26.0 and 25.0 °C in 2018 and 2019 and winter temperatures of 9.1 °C and 10.8 °C, and mean rainfall of 2018 (143.2 mm) and 2019 (157.1 mm), respectively (see Table 2 in [41] for details).

Pots at 57 cm × 23 cm × 27 cm (length × width × height) were filled with 26 kg purple soil that has developed from –brown–purple sand shale parent materials. This soil is classified as Eutric Regosol according to the FAO Soil Classification System [68]. Soil samples at 0–20 cm depth were collected at the aforementioned NMBPSFFE. The soil was characterized by a texture of sand:silt:clay (14%:48%:38%), 1.37 g cm^−3^ bulk density, pH (1:2.5; H_2_O) of 6.9, 7.40 g soil organic carbon kg^−1^, 0.70 g total N kg^−1^, 81.90 mg available N kg^−1^, 0.42 g total P kg^−1^, 15.70 mg available P kg^−1^, 10.16 g total K kg^−1^, and 176 mg available K kg^−1^.

Two maize hybrid cultivars of Xida-789 and Xida-211 were respectively planted in 2018 and 2019. Maize seeds were surface-sterilized using 10% H_2_O_2_ for 20 min, followed by thorough rinsing with sterile water, and then pre-germinated on sterilized moist paper at 20/25 °C (night/day) for 1.5 days. Each pot was initially sown with five seeds, but after ten days of germination, only two healthy seedlings were retained per pot. Plants were observed daily throughout the growing season, and soil moisture was maintained at 70% field water-holding capacity. Weeding was carried out at 15 and 45 days after sowing (DAS). The Philippine downy mildew, which typically occurs during the maize’s tasseling stage as identified by pale yellow to whitish discolorations, was controlled with a diluted 1/1000 solution of 25% Metalaxyl.

### 4.2. Experimental Design and Treatments

A randomized split-plot design was employed for the experimentation. The main factor was the fertilizer placement method: broadcasting; side banding; or deep banding. The sub-factor was a combination of N and P fertilizer rates of 112, 150, 187 kg N ha^−1^ and 45, 60, 75 kg P ha^−1^, resulting in a total of nine combinations of NP fertilization treatments: N112P45; N112P60; N112P75; N150P45; N150P60; N150P75; N187P45; N187P60; and N187P75, while no-NP was the control treatment. The growth pot had a capacity to hold 26 kg of air-dried soil. The fertilizer placement methodology was applied as follows: (1) Broadcast: A mixture of 5 kg soil plus required N and P fertilization rates were evenly spread on the soil surface; (2) Side band: A mixture of 0.5 kg soil plus required N and P fertilization rates were buried as a 4 cm narrow strip on the soil surface with a 7 cm distance from the sowing line; and (3) Deep band: A mixture of 0.5 kg soil plus required N and P fertilization rates were buried as 4 cm narrow strip at 7 cm depth with a 7 cm distance from the sowing line. The commercial urea (46% N) and calcium superphosphate (P_2_O_5_ ≥ 12%) were utilized as basal fertilizers in a single application prior to seeding, in addition to potassium chloride (40 kg ha^−1^, 52% K) to ensure a balanced NPK supply. Each fertilization treatment was replicated thrice for a total of 30 pots or replicates (see Figure 11 in [41] for details).

### 4.3. Harvesting and Phosphorus Variables Determination

Aboveground leaves and stems at three stages were harvested in the eight-leaf stage (V8 at 45 DAS), tasseling stage (VT at 60 DAS), and physiological maturity or harvest stage (R6 at 115 DAS). Roots were collected at VT and R6, and seeds were also harvested at R6. Harvested plant tissues were washed carefully with tap water and then oven-dried at 60 °C to a constant weight.

Plant samples were ground to powder, passed through a 1 mm sieve, and then digested with 98% sulfuric acid and 30% hydrogen peroxide. After digestion, tissue P concentrations were measured using the vanadium molybdate yellow colorimetric method [69].

The concentrations of total P and Olsen-P in the soil were measured at 60 DAS and 115 DAS. To determine total soil P, 0.20 g of air-dried soil samples were weighed into a 30 mL nickel crucible and heated in an electric furnace at 700–720 °C for 15 min after the addition of 2.0 g sodium hydroxide. After cooling, the digested mixture was dissolved in deionized water, and the total volume was brought up to 100 mL. Total soil P concentration was determined using the colorimetric molybdate and ascorbic acid method [70], with the absorbance measured at 660 nm. Soil Olsen-P was determined using the molybdovanado phosphatase method based on the extraction with 0.5 M NaHCO_3_ [71]. Plant tissue P accumulation was calculated by multiplying P concentrations in leaves, stems, seeds, and roots with their respective biomasses. Total plant P accumulation was calculated as the sum of P accumulation in leaves, stems, seeds, and roots.

(1) $$\mathrm{Agronomy}\;P\;\mathrm{use}\;\mathrm{efficiency}=\frac{\mathrm{seed}\;\mathrm{yield}\;\mathrm{at}\;P\;\mathrm{treatment}\;-\;\mathrm{seed}\;\mathrm{yield}\;\mathrm{at}\;\mathrm{zero}\;P\;\mathrm{treatment}\;}{\mathrm{applied}\;P\;\mathrm{at}\;P\;\mathrm{treatment}}$$

(2) $$\mathrm{Phosphorus}\;\mathrm{use}\;\mathrm{efficiency}\;(\%)=\frac{(U_{P}-U_{0})\;}{A_{P}}\;\times \;100$$

where U_P_ or U_0_ is total P accumulation (leaf + stem + seed + root) under a specific P or no-P fertilization (control), and AP is the total applied P from the specific P fertilization.

Partial factor productivity of P fertilizer (PFP_P_):

(3) $$\mathrm{PFP}P\;(\mathrm{kg}\;\mathrm{grain}\;{\mathrm{kg}}^{-1}\;P)=\frac{Y_{P}}{A_{P}}$$

where Y_P_ is the grain yield, and A_P_ is the total P accumulation under a specific P fertilization.

### 4.4. Statistical Analyses

Data (means ± SE, *n* = 6) for each of all the measured parameters were combined and averaged under three fertilizer placements for the control treatments and under the same N and P fertilizers for the same fertilizer placement since no significant differences had been observed between maize varieties and years (2018 and 2019). Statistical analyses were performed using SPSS 24.0 software (SPSS Inc., Chicago, IL, USA). The data were checked for normality and homogeneity of variance using Kolmogorov–Smirnov and Shapiro–Wilk tests. Two-factor analysis of variance (ANOVA) was used to assess the effect of fertilizer placement methods and different N and P fertilization rates. One-way ANOVA was performed to compare the mean effect of a single factor (fertilizer placement methods) and the same N or P with different P or N fertilization rates. Data comparisons among treatments were examined by Duncan’s multiple range test at *p* < 0.05. Correlations between fertilizer placement methods and fertilization rates were conducted using OriginPro2023b version 10.05 (Origin Lab Corp., Northampton, MA, USA).

## 5. Conclusions

Deep band placement significantly enhances P uptake efficiency, leading to greater P concentration and accumulation in maize leaves, stems, roots, and seeds compared to the other two methods. Higher N application rates (N187 and N150), when combined with deep band placement, further promote the mechanisms that enhance P availability and utilization by the plant. Deep band placement also led to significantly increased total soil P concentration, Olsen-P concentration, and P use efficiency compared to other placement methods. This suggests that deep band placement is a more efficient strategy for P fertilization in maize. It may allow for reduced P application rates while achieving similar or improved growth and yield outcomes. This study found positive relationships between P concentration and accumulation in various plant organs and soil Olsen-P concentration. This highlights the importance of maintaining adequate soil P levels for optimal plant growth. In conclusion, this study suggests that deep band fertilizer placement is a superior strategy for enhancing P uptake efficiency, utilization, and overall productivity in maize compared to broadcast and side band placement methods. The approach and outcome from the deep band fertilization in this greenhouse study can be advocated for field practices to optimize P fertilizer use and improve maize production while minimizing potential environmental P losses associated with broadcast application.

## Figures and Tables

**Table 1:** Effects of nitrogen (N) and phosphorus (P) fertilization rates and fertilizer placement on phosphorus agronomy efficiency (PAE, kg kg^−1^), P use efficiency (PUE, %), and partial factor productivity of phosphorus (PFPP, kg kg^−1^ P) of maize.

NP Rate	Placement	PAE (kg kg^−1^)	PUE (%)	PFP_P_ (kg kg^−1^ P)
N112P45	Broadcast	35.4 ± 9.2 (a,x,e)	7.7 ± 1.3 (b,x,e)	76.4 ± 5.4 (a,x,e)
	Side band	34.3 ± 5.7 (a,x,e)	8.0 ± 0.6 (a,x,e)	63.9 ± 3.0 (a,y,e)
	Deep band	23.3 ± 6.0 (a,x,e)	6.2 ± 1.0 (a,x,e)	48.9 ± 2.8 (a,z,e)
N150P45	Broadcast	37.3 ± 4.8 (a,x,e)	8.9 ± 1.1 (a,x,e)	78.0 ± 4.4 (a,x,e)
	Side band	38.5 ± 7.5 (a,x,e)	11.3 ± 1.7 (a,x,e)	81.3 ± 3.0 (a,x,e)
	Deep band	43.5 ± 9.1 (a,x,e)	13.6 ± 1.9 (a,x,e)	84.1 ± 5.9 (a,x,e)
N187P45	Broadcast	39.3 ± 8.2 (a,x,e)	11.5 ± 1.7 (a,x,e)	79.8 ± 4.6 (a,x,e)
	Side band	42.3 ± 7.7 (a,x,e)	13.6 ± 1.6 (a,x,e)	84.5 ± 6.6 (a,x,e)
	Deep band	45.9 ± 7.5 (a,x,e)	15.6 ± 1.4 (a,x,e)	86.1 ± 3.2 (a,x,e)
N112P60	Broadcast	37.1 ± 7.2 (a,x,e)	10.0 ± 1.4 (ab,x,f)	80.1 ± 3.0 (a,x,e)
	Side band	34.4 ± 7.8 (a,x,e)	9.0 ± 1.4 (a,x,e)	65.6 ± 3.4 (a,y,e)
	Deep band	25.9 ± 6.0 (a,x,f)	7.8 ± 1.1 (a,x,f)	51.0 ± 3.6 (a,z,f)
N150P60	Broadcast	36.2 ± 4.4 (a,x,e)	9.1 ± 0.9 (a,y,ef)	65.5 ± 3.9 (b,y,e)
	Side band	38.3 ± 4.4 (a,x,e)	10.7 ± 1.0 (a,y,e)	69.0 ± 4.2 (b,xy,e)
	Deep band	49.5 ± 4.5 (a,x,ef)	15.2 ± 1.2 (a,x,e)	77.6 ± 3.2 (a,x,e)
N187P60	Broadcast	37.3 ± 3.5 (a,y,e)	10.3 ± 0.5 (a,y,e)	66.5 ± 3.2 (b,y,e)
	Side band	40.5 ± 5.4 (a,xy,e)	12.5 ± 1.0 (a,y,e)	70.9 ± 4.5 (a,xy,e)
	Deep band	51.5 ± 3.5 (a,x,e)	17.0 ± 0.9 (a,x,e)	79.2 ± 3.4 (a,x,e)
N112P75	Broadcast	41.7 ± 8.0 (a,x,e)	11.7 ± 1.2 (a,x,e)	82.5 ± 4.4 (a,x,e)
	Side band	36.9 ± 5.6 (a,x,e)	10.2 ± 1.2 (a,x,f)	66.7 ± 3.1 (a,y,e)
	Deep band	31.4 ± 6.6 (a,x,e)	9.1 ± 0.8 (a,x,f)	53.5 ± 3.2 (a,z,f)
N150P75	Broadcast	25.2 ± 6.0 (a,y,e)	7.6 ± 0.9 (a,z,e)	50.5 ± 3.5 (c,y,e)
	Side band	27.4 ± 2.5 (a,y,e)	9.5 ± 0.4 (a,y,ef)	52.3 ± 3.2 (c,y,e)
	Deep band	44.0 ± 3.0 (a,x,e)	14.5 ± 0.5 (a,x,e)	64.4 ± 3.2 (b,x,e)
N187P75	Broadcast	25.9 ± 4.1 (a,y,e)	8.5 ± 1.0 (a,z,e)	51.1 ± 2.8 (c,y,e)
	Side band	31.0 ± 2.2 (a,y,e)	10.8 ± 0.4 (a,y,e)	55.4 ± 3.5 (b,y,e)
	Deep band	46.3 ± 4.6 (a,x,e)	15.9 ± 0.4 (a,x,e)	66.4 ± 3.9 (b,x,e)

## Data Availability

Data are contained within this article.
